# Adolescents in Quarantine During COVID-19 Pandemic in Italy: Perceived Health Risk, Beliefs, Psychological Experiences and Expectations for the Future

**DOI:** 10.3389/fpsyg.2020.559951

**Published:** 2020-09-23

**Authors:** Elena Commodari, Valentina Lucia La Rosa

**Affiliations:** Department of Educational Sciences, University of Catania, Catania, Italy

**Keywords:** COVID-19, quarantine, adolescents, health risk, Italy

## Abstract

Since March 2020, many countries throughout the world have been in lockdown in response to the COVID-19 pandemic. In Italy, the quarantine began on March 9, 2020, and containment measures were partially reduced only on May 4, 2020. The quarantine experience has a significant psychological impact at all ages but can have it above all on adolescents who cannot go to school, play sports, and meet friends. In this scenario, this study aimed to provide a general overview of the perceived risk related to COVID-19 and the psychological experience of quarantine in a large sample of Italian adolescents. Nine hundred and seventy eight adolescents (males = 339; females = 639) living in 13 Italian regions and attending upper secondary school (age range: 13–20, *M* = 16.57, *SD* = 1.20), responded to an internet-based questionnaire about perceived health risk related to COVID-19, knowledge and information on measures to control the pandemic, beliefs and opinions on stage two of the quarantine, and psychological experiences related to quarantine. 31.1% of the participants lived in “red zones,” which are places where the government has imposed stricter measures of containment due to exponential and uncontrolled growth in contagion cases compared to other areas in Italy. According to our results, Italian adolescents had a low perception of risk of COVID-19. Perceived comparative susceptibility and perceived seriousness were also very low. However, they were aware of the restriction measures necessary to contain the spread of the virus, and they agreed with the limitations imposed by the government. Females and adolescents living in a “red zone” showed more significant psychological negative feelings about the quarantine experience. However, no significant differences were found about the regions where the teenagers of our sample live and the other variables related to the COVID-19 experience. This is very interesting data, leading us to hypothesize that the participants’ negative feelings may be more related to the adolescent period than to the pandemic itself.

## Introduction

Since March 2020, many countries throughout the world have been in lockdown in response to the COVID-19 pandemic. In Italy, the quarantine began on March 9, 2020, and containment measures were partially reduced only on May 4, 2020. People had to stay at home. All social and sporting activities were canceled, and many work activities were forbidden; schools were closed and will reopen only with the new academic year.

Many countries around the world have temporarily closed educational institutions to contain the spread of the COVID-19 pandemic. According to the United Nations Educational, Scientific and Cultural Organization (UNESCO), school closures have impacted over 90% of the world’s student population (UNESCO, 2020). Italy and several other countries have used educational technologies, including online platforms, radio, television, and texting, to support access to remote learning during the COVID-19 pandemic, and so guarantee the students’ right to education (UNESCO, 2020).

Changes in life caused by the pandemic were dramatic for people of all ages. However, the revolution of behavioral routines caused by quarantine can have been particularly hard to accept for young people who could not go to school, play sports, and meet friends. Usually, adolescents spend much of their waking time in school or other social contexts, such as gyms or recreational spaces (Mahoney et al., 2009). Now they have had to stay home all day for months, with online relationships only with peers and adults, such as their teachers, except the persons that live with them. Moreover, their home has become a “school.” For these reasons, there was an extensive debate between scientists on the effects of quarantine, limitations of freedom, and school closures on adolescents’ emotional and affective states.

Previous studies on the effect of quarantine, which is the separation and restriction of movement of people who can be potentially exposed to a contagious disease, report common psychological effects (Brooks et al., 2020). Research conducted on the Severe Acute Respiratory Syndrome (SARS) epidemic (Hawryluck et al., 2004; Mihashi et al., 2009; Liu et al., 2012) reported a high prevalence of symptoms of psychological distress, such as insomnia, irritability, anger, and other mood disorders. However, although the risk for psychological disorders related to changes in life caused by an epidemic is largely documented, there is evidence that several sociodemographic and psychological variables influence the emotional responses to behavioral limitations (Hawryluck et al., 2004). In particular, the perceived health risk for disease affects the emotional responses to the prevention measures and the acceptance of limitations of behavior (Tang and Wong, 2003; Commodari, 2017).

Health-related perceived risk depends on perceived “seriousness” and perceived “susceptibility” to a disease. Risk perception is one of the key drivers of health behavior (Brewer et al., 2004; Ibuka et al., 2010; Commodari et al., 2020) and influences the adoption of precautionary measures. Perceived seriousness refers to how at risk a person considers himself to develop a disease, while perceived susceptibility concerns the perceived probability of getting a disease. Perceived susceptibility can be differentiated into perceived personal susceptibility, which is the perceived probability that one will be harmed by a hazard (Rogers, 1983), and perceived comparative susceptibility, which is the perceived probability that a hazard will hurt one compared with other people of the same age and gender.

Research on health-related risk perception in young people has shown that adolescents engaged in risky behavior do not have a complete appreciation of their exposure to harm (Johnson et al., 2002). However, there are no previous studies on the health risk perception for pandemic diseases and the psychological experiences related to quarantine in this stage of the life span.

### Study Aim and Hypotheses

Based on these considerations, the main goal of this study was to investigate the perceived risk related to COVID-19 and the psychological experiences of adolescents during the pandemic. In particular, the purpose of the study was to analyze the perceived seriousness of and susceptibility to COVID-19, the beliefs of adolescents in the first phase of quarantine and their opinions on the stage two of quarantine, during which a partial reduction of behavioral measures was hypothesized. Moreover, the study explored adolescents’ moods, emotions, and feelings, with attention to expectations for the immediate future. More specifically, the study intended to verify the following hypotheses:

Hypothesis 1 (H1): Living in an area with more restrictions than in other areas of the country contributes significantly to increasing the disease’s perception of risk.
Hypothesis 2 (H2): Living in an area with more restrictions than in other areas of the country significantly contributes to accentuating the negative psychological impact of the quarantine experience.
Hypothesis 3 (H3): Other sociodemographic variables influence health risk perception and psychological experiences of the adolescents in the sample. In particular, a higher perception of risk and a greater concern of contracting COVID-19 were expected to predict more negative feelings during the quarantine. Conversely, it was expected that higher adherence to government measures to contain the infection and greater confidence in the information received on COVID-19 were predictors of positive emotions.

## Materials and Methods

### Participants

Participants were 978 adolescents (males = 339; females = 639) who attended upper secondary school (age range: 13–20, *M* = 16.57, *SD* = 1.20), which corresponds to the International Standard Classification of Education Level 3. The participants lived in 13 of the 20 Italian regions which are the first-level constituent entities of the Italian Republic. Five hundred and seventy four of the respondents lived in a provincial seat, while 404 lived in towns that are not the provincial seat. Teachers and some students collaborated in the recruitment of the participants, sharing an online survey on the leading social networks and inviting students to respond to the questionnaire.

### Measures

Data were collected using an internet-based questionnaire. In total, the questionnaire consisted of 81 multiple-choice and open-ended questions. Participation was voluntary, and the questionnaire required approximately 10–15 min to complete. The survey collected sociodemographic information, such as age, gender, area in which the respondents live, type of upper secondary school, academic grade, number of persons in the household, and other information. Moreover, it explored perceived health risk related to COVID-19, knowledge and information on measures to control the pandemic, beliefs and opinions on stage two of the quarantine, routines and habits of life that adolescents miss most (such as going out with friends, meeting boyfriend or girlfriend, going to visit their relatives, for a total of six items), and psychological experiences related to quarantine. The survey also collected information on e-learning experiences during quarantine, but this subject is beyond the scope of this article.

Perceived seriousness, perceived personal susceptibility, and perceived comparative susceptibility to COVID-19, which are the main dimensions of risk perception related to health, were investigated using an adjustment of the Italian version (Commodari, 2017) of the Risk Perception of Infectious Diseases Questionnaire (Brug et al., 2004). Participants responded to questions using a five-point Likert-type scale. The participants were invited to report (a) how serious it would be for them to get the disease, (b) how likely they think they are to contract the disease, (c) whether they would have a smaller or larger chance of getting the disease before summer, compared with their peers of the same age and gender, and (d) if they believed that students could be a category particularly at risk of contracting the virus.

The original version of the Risk Perception of Infectious Diseases Questionnaire was developed during the SARS epidemic, and it was translated into several languages (de Zwart et al., 2010). Its psychometric characteristics are good (Cronbach’s α = 0.79), and many international studies have used this measure in different contexts (de Zwart et al., 2009, 2010; Commodari, 2017). In this regard, a recent study by Commodari (2017) used the Italian adjustment of this questionnaire to investigate the risk perception of flu and the role of sociodemographic and psychological variables on perceived risk. A confirmatory factor analysis was run to assess the validity of this adapted version and a good model fit was obtained [χ^2^(5) = 21.5; *p* < 0.001; RMSEA = 0.05; SRMR = 0.03; CFI = 0.945; TLI = 0.95]. The reliability is also good (Cronbach’s α = 0.80).

Regarding their opinions and beliefs, participants were asked to indicate whether they agreed with statements of reported information on COVID-19 and quarantine (e.g., “There are some categories of people at higher risk for COVID-19 than the general population”; “In stage two of the quarantine it is necessary to avoid the use of public transport to reduce the risk of contagion and to avoid a new increase in the epidemic,” and others). Regarding feelings, emotions, and moods, participants were asked to complete a Likert-type scale that focused on the personal feelings about one’s cognitive, physiological, and behavioral state. Participants indicated their level of agreement with several statements using a five-point Likert-type scale (e.g., “In this period in which I have to stay at home, I feel well physically”; “In this period in which I have to stay home I am tense and I feel tight”). The scale measured two aspects: “negative feelings” and “positive feelings.” A high score corresponded to high perception of negative or positive feelings, respectively. A CFA was also performed to assess the validity of these scores. Regarding the model for the “negative feelings,” although the Chi-square statistic resulted to be statistically significant [χ^2^(27) = 165; *p* < 0.001], the other values were indicative of a good model fit (RMSEA = 0.07; SRMR = 0.04; CFI = 0.943; TLI = 0.95). The same result was obtained also for the model of the “positive feelings” [χ^2^(9) = 40.6; *p* < 0.001; RMSEA = 0.05; SRMR = 0.03; CFI = 0.932; TLI = 0.96]. Both the scales showed a good reliability (negative feelings: Cronbach’s α = 0.81; positive feelings: Cronbach’s α = 0.78). The scores were converted into *z* scores for the purpose of statistical analyses.

Finally, participants were asked to answer questions about their routines and habits of life they missed most, and to complete, without a word limit, the sentence “In this period in which I have to stay home, I think my summer will be…”.

### Procedures

Participants completed the online survey between April 22 and May 1, 2020. The Statistical Package for the Social Sciences (SPSS) version 25.0 (IBM Corporation, Armonk, NY, United States) was used for the statistical analyses. Quantitative data were expressed as frequencies and percentages in the case of categorical and ordinal variables and as mean and standard deviation in the case of continuous variables. Independent-samples *t*-test were run and the magnitude of the differences between the means were assessed using Hedges’s *g* formula (Hedges, 1981) to calculate effect size (ES), with 0.20 indicating a small ES, 0.50 a medium ES, and 0.80 a large ES (Cohen, 1988). Besides, two multiple regression analyses were calculated to investigate the impact of sociodemographic variables and risk perception on positive and negative feelings experienced during the lockdown. For this purpose, the main sociodemographic variables and risk perception values were the independent variables, and the “positive feelings” and “negative feelings” scores were the dependent variable.

Qualitative data were coded and analyzed to show emerging themes. The thematic coding structure’s development and confirmation was an iterative process involving two researchers conducting individual, recursive reading of the textual data and group meetings to discuss and test the emerging themes. Discrepancies were resolved by consulting specific instances in the data, discussing their relationship to establish themes, and reaching consensus as a group (Corbin and Strauss, 2014). The participants’ responses to some items were free, and these could be single words or full sentences. Codification was realized using “thought unit,” also denoted “sense unit” or “unit of meaning.” The units comprised one idea communicated, whether it was expressed as a sentence, a verb-object sequence, or a single word. The responses were first categorized into 55 categories. Then, these categories were progressively reduced. Finally, the responses were coded in 52 categories (Srnka and Koeszegi, 2017).

The study was performed following the ethical standards of the 1964 Declaration of Helsinki and followed the Ethical Code for Italian psychologists (L. 18.02.1989, n. 56), Italian law for data privacy (DLGS 196/2003), and the Ethical Code for Psychological Research (March 27, 2015) approved by the Italian Psychologists Association. No sensitive data that could identify the participants was collected. The schools involved in the research had previously informed the students’ parents to consent to the study’s participation. The Chair of School and Family Psychology, DISFOR, University of Catania, approved this study.

## Results

### Sociodemographic Characteristics of the Sample and Diffusion of COVID-19 Among Adolescents

Table 1 shows the sociodemographic characteristics of the participants. Participants lived in 13 of the 20 Italian regions. COVID-19 was not diffused equally throughout Italy. The regions in which participants lived reported on May 3 different levels of contagion, with a broader spread of the virus in the regions of Northern Italy. Of the student participants, 31.1% lived in “red zones,” which were places in which the government has imposed stricter measures of containment due to an exponential and uncontrolled growth in cases of contagion compared to other areas in Italy. Regarding the number of persons with whom respondents were spending quarantine, 20.1% reported that the size of their household was more than four people, including themselves; 46.5% reported four persons; 24.9%, three persons; 7.6%, two persons; and 0.8% reported living alone during the quarantine. Five of the respondents (0.5%) had been or was currently suffering from COVID-19, while 0.8% of the respondents reported that they were uncertain about having had this disease; 3.1% of respondents reported that one or more family members living with them had been or was currently suffering from COVID-19. Furthermore, 10.2% of the sample had at least one family member who worked with people affected by COVID-19 (health care or other essential services).

**TABLE 1:** Socio-demographic characteristics of the sample.

		*N*	%
Gender	Female	639	65.3
	Male	339	34.7
Age (years old)	13	7	0.7
	14	58	5.9
	15	96	9.8
	16	303	31.0
	17	284	29.0
	18	195	19.9
	19	27	2.8
	20	8	0.8
School	High school	782	80.0
	Technical institute	148	15.1
	Professional institute	48	4.9
Town	Chief town	574	58.7
	Not chief town	404	41.3
Regions	Lombardy	44	4.5
	Piemont	11	1.1
	Trentino Alto Adige	2	0.2
	Friuli Venezia Giulia	2	0.2
	Emilia Romagna	22	2.2
	Abruzzo	11	0.5
	Molise	7	0.7
	Toscana	9	0.9
	Umbria	44	4.5
	Lazio	337	34.5
	Campania	80	8.2
	Puglia	17	1.7
	Sicily	398	40.7
“Red zone”	Yes	304	31.1
	No	674	68.9
Size of the household	1	8	0.8
	2	74	7.6
	3	244	24.9
	4	455	46.5
	>4	197	20.1
Affected by	Yes	5	0.5
COVID-19	No	965	98.7
	Uncertain	8	0.8
Family member	Yes	30	3.1
with COVID-19	No	948	96.9
Family member working	Yes	100	10.2
with COVID-19 people	No	878	89.8

### Perceived Health Risk

Table 2 reports data on the perceived health risk in the sample. In general, adolescents considered the possibility of getting COVID-19 before summer to be low: 60.3% responded that their probability was very low or low, while only 4.5% think that this probability was high or very high (27.7% very low; 32.6% low; 33.9% neither low nor high; 3.7% high; and 0.8% very high). Furthermore, most subjects believed they had a low or very low probability of contracting the virus before the summer compared to peers of the same age and gender (20.7% very low; 33.7% low; 39.7% neither low nor high). Only 4.3% of the sample thought that this probability was high and 0.9% very high.

**TABLE 2:** Frequencies and percentages of the perceived seriousness, personal and comparative susceptibility to COVID-19 in the sample.

	Perceived personal susceptibility		Comparative susceptibility		Perceived seriousness	
	*n*	%	*n*	%	*n*	%
No answer	12	1.2	7	0.7	5	0.5
Very low	271	27.7	202	20.7	37	3.8
Low	319	32.6	330	33.7	182	18.6
Neither low nor high	332	33.9	388	39.7	304	31.1
High	36	3.7	42	4.3	297	30.4
Very high	8	0.8	9	0.9	153	5.6

Regarding the perceived seriousness of the disease, 36% of the sample believed that contracting the virus could be serious or very serious (30.4% serious and 5.6% very serious), and 31.1% believed that it was neither serious nor not serious. Only a limited percentage believed that getting COVID-19 was not at all or not very serious (3.8% not at all serious and 18.6% not very serious). The respondents who lived in a red zone presented a higher perceived risk compared to those that live in other places of Italy with an ES approaching to large (red zone: *M* = 2.75, *SD* = 0.91; non-red zone: *M* = 2.09, *SD* = 0.89; *t* = 3.55, *p* < 0.001; *g* = 0.74). Furthermore, females showed a higher perceived risk than males with a medium ES (females: *M* = 2.54, *SD* = 0.90; males: *M* = 2.08, *SD* = 0.89; *t* = −1.99; *p* = 0.04; *g* = −0.51).

There were no differences by regions in perceived health risk, although some of these regions were the most affected by the disease. Moreover, the presence of parents or other family members who had been or was currently suffering from COVID-19 did not influence risk perception. Interestingly, both the perceived susceptibility (family member working with people who had COVID-19: *M* = 2.61, *SD* = 0.92; no family member working with people who had COVID-19: *M* = 2.13, *SD* = 0.89; *t* = 3.36; *p* = 0.001; *g* = 0.53) and comparative susceptibility (family member working with people who had COVID-19: *M* = 2.71, *SD* = 0.97; no family member working with people who had COVID-19: *M* = 2.26, *SD* = 0.85; *t* = 4.91; *p* < 0.001; *g* = 0.52) were higher in those adolescents whose parents or other relatives worked with persons sick with this disease and the ES was medium. To better analyze the perception of the risk for COVID-19, students were also invited to report how frightened they were of getting COVID-19. The majority of the sample was not particularly afraid of contracting COVID-19. There were no significant differences in the responses of the students by region. The students who lived in the regions with higher diffusion of the disease did not show greater fear of the disease than their peers. However, the students who lived in a red zone were more fearful of COVID-19 than their peers with a medium ES (red zone: *M* = 2.91, *SD* = 1.32; non-red zone: *M* = 2.22, *SD* = 1.20; *t* = 2.71; *p* = 0.007; *g* = 0.55) (Table 3).

**TABLE 3:** Differences in health risk perception according to socio-demographic variables.

	Male (*n* = 337)		Female (*n* = 629)		*t-test*	*g*	“Red zone” (*n* = 301)		Not “Red zone” (*n* = 665)		*t-test*	*g*	Family member working with COVID-19 people (*n* = 100)		Family member not working with COVID-19 people (*n* = 866)		*t-test*	*g*
												
	*M*	*SD*	*M*	*SD*			*M*	*SD*	*M*	*SD*			*M*	*SD*	*M*	*SD*		
Perceived susceptibility	2.08	0.89	2.54	0.90	−1.99*	−0.51	2.75	0.91	2.09	0.89	3.55**	0.74	2.61	0.92	2.13	0.89	3.36**	0.53
Perceived comparative susceptibility	2.29	0.87	2.31	0.87	−0.29	−0.02	2.37	0.87	2.28	0.87	1.55	0.10	2.71	0.97	2.26	0.85	4.91**	0.52
Perceived seriousness	3.29	1.11	3.39	1.04	−1.41	−0.09	3.41	1.10	3.33	1.05	1.07	0.07	3.24	1.24	3.37	1.04	−1.00	−0.12
Fear of getting COVID-19	2.20	1.21	2.35	1.25	−1.723	−0.12	2.91	1.32	2.22	1.20	2.71**	0.55	2.34	1.20	2.29	1.24	0.36	0.04

*p < 0.05; **p < 0.01. M, mean; SD, standard deviation; g, Hedge’s g.

### Knowledge and Opinions on COVID-19 and Stage Two of Quarantine

Ninety three percent of respondents believed that there were categories of people more at risk of getting COVID-19, but, interestingly, 76.2% of the respondents did not consider students as a category at risk for COVID-19. The remaining participants (21.9%) believed that students were an at-risk category for this disease. These students motivated their response with the argument that the school setting does not permit social distancing. The remaining 1.9% did not answer the question.

A significant percentage of the respondents reported having confidence in the information that they received on the disease (56.1% trust enough; 18.3% trust a lot; 0.2% trust very much). Moreover, the most critical information the adolescents would have liked to receive on COVID-19 concerned on how to cure the disease (42.8% of the respondents). Interestingly, only 17.2% of respondents were interested in how to prevent the infection. Furthermore, 11.2% wanted information on the likelihood of contracting the virus in an area of residence, 10.6% how to recognize the symptoms of the disease, 3% the geographical areas where the virus is most present, and only 0.2% would have liked to have been more informed about how the virus was transmitted.

Regarding adolescents’ opinions of the behavioral measures that could be useful to maintain in stage 2 of quarantine, during which there was a partial reduction of containment measures, a very high percentage of respondents (89.1%) agreed on the need to avoid public transport, such as trains or busses, as well as to confined spaces such as bars, restaurants, cinemas, theaters, and school classrooms (91.8%). Similarly, 90.2% of the respondents agreed with the need to avoid going into shops if not necessary and only with personal protective equipment, such as a face mask. Further, 84.4% of the respondents agreed with the need to avoid going to gyms or swimming pools, and 72.5% considered it useful to avoid medical consultations if possible. However, adolescents did not think it will be necessary to maintain social distancing in the second quarantine stage. Most respondents did not agree on the need to avoid staying with persons who are not cohabiting (57.0%), and 82.6% think that it is not necessary to avoid staying in open places such as parks. Data are summarized in Table 4.

**TABLE 4:** Adolescents’ opinions on the behavioral measures to maintain in the stage 2 of the quarantine.

	Yes		No	
	*n*	%	*n*	%
Avoid using public transport (trains, busses, planes)	871	89.1	107	10.9
Avoid going to closed places such as bars, restaurants, cinemas and theaters, classrooms	898	91.8	80	8.2
Avoid going to shops if not necessary and with the necessary protections (facial mask)	882	90.2	96	9.8
Avoid meeting non-cohabiting people	42	43.0	557	57.0
Avoid unnecessary medical visits	709	72.5	269	7.5
Avoid walking in open places	170	17.4	808	82.6
Avoid playing sports in gyms or swimming pools	825	84.4	153	15.6

Interestingly, adolescents showed a high awareness of the particularity of the moment in which Italy was living. The vast majority of the sample reported having no difficulty complying with the government’s restrictive provisions (73.1%), and they substantially agreed with the restrictions imposed on citizens due to the pandemic (81.9%).

### Daily Routines of Adolescents During the Quarantine

The majority of the adolescents interviewed said they had more homework than before due to the remote school activities (66.8%), and a significant percentage of them (40.5%) reported having little free time. However, a large percentage was coping with quarantine by dedicating at least 1 h a day to a hobby (61.5%), watching television or playing video games (42.4%), or spending much more time on social networks such as Facebook or Instagram (62.3%). More specifically, males tended to devote themselves to hobbies (males: *M* = 3.84, *SD* = 1.38; females: *M* = 3.53, *SD* = 1.42; *t* = 3.29, *p* = 0.001; *g* = 0.22) and to watching TV or playing video games (males: *M* = 3.49, *SD* = 1.27; females: *M* = 2.81, *SD* = 1.40; *t* = 7.62; *p* < 0.001; *g* = 0.50), while females spent more time on social networks (females: *M* = 3.83, *SD* = 1.18; males: *M* = 3.30, *SD* = 1.31; *t* = −2.74; *p* = 0.006; *g* = −0.43). These differences were small or approaching to medium. Furthermore, adolescents living in a red zone tended to watch TV or play video games more than peers who did not live in a red zone but the difference was small (red zone: *M* = 3.20, *SD* = 1.34; no red zone: *M* = 2.97, *SD* = 1.41; *t* = 2.44; *p* = 0.015; *g* = 0.16) (Table 5).

**TABLE 5:** Comparison of the answers to the questionnaire according to gender and residence in a “red zone.”

	Male (*n* = 337)		Female (*n* = 629)		*t-test*	*g*	Living in a “red zone” (*n* = 301)		Not living in a “red zone” (*n* = 665)		*t-test*	*g*
								
	*M*	*SD*	*M*	*SD*			*M*	*SD*	*M*	*SD*		
In this period in which I have to stay at home I feel physically well	3.32	1.17	2.97	1.16	4.54**	0.30	2.99	1.18	3.14	1.17	−1.81	−0.12
In this time when I have to stay home, I get bored listening to other people’s problems	2.89	1.16	2.53	1.28	4.54**	0.29	2.69	1.36	2.64	1.20	0.59	0.04
In this time when I have to stay home, I frankly express my emotions	3.19	1.20	3.13	1.27	0.66	0.04	3.18	1.26	3.13	1.24	0.58	0.04
In this time when I have to stay at home, I feel confident in myself	3.18	1.14	2.50	1.23	8.58**	0.56	2.75	1.20	2.73	1.26	0.30	0.01
In this time when I have to stay home, I feel tense or I feel tight	2.87	1.26	3.40	1.27	−6.22**	−0.41	3.23	1.34	3.21	1.27	0.14	0.01
In this time when I have to stay home, I feel my heart beat faster or irregularly	1.81	1.17	2.33	1.34	−6.33**	−0.40	2.72	1.38	2.07	1.26	2.82**	0.50
In this period in which I have to stay at home, I have difficulty falling asleep	3.15	1.48	3.60	1.40	−4.67**	−0.31	3.73	1.44	3.37	1.44	2.45*	0.25
In this time when I have to stay at home, I always think of the same things and feel my head full of thoughts	3.30	1.31	3.83	1.18	−6.13**	−0.43	3.72	1.26	3.61	1.24	1.26	0.08
In this period when I have to stay at home, I am irritable and I lose patience	2.95	1.38	3.53	1.36	−6.36**	−0.42	3.98	1.37	3.25	1.40	2.70**	0.52
In this time when I have to stay home, I am discouraged, depressed, downcast	2.65	1.38	3.29	1.37	−6.98**	−0.46	3.35	1.46	3.00	1.38	2.21*	0.25
In this time when I have to stay at home, I feel like crying more frequently than usual	1.86	1.20	3.18	1.50	−14.93**	−0.94	2.86	1.56	2.66	1.52	1.91	0.13
In this period in which I have to stay at home, I especially miss not meeting my friends	3.74	1.35	3.33	1.47	4.32**	0.28	3.50	1.45	3.46	1.44	0.35	0.02
In this period in which I have to stay at home, I especially miss not meeting my relatives	3.58	1.29	3.80	1.25	−2.58*	−0.17	3.79	1.28	3.69	1.26	1.11	0.07
In this period when I have to stay at home, I spend at least an hour a day playing a musical instrument, dancing, gymnastics, acting, drawing, or doing the things I like	3.84	1.38	3.53	1.42	3.29**	0.22	3.63	1.45	3.64	1.40	−0.15	−0.007
In this time when I have to stay at home, I spend more than half of my day fantasizing	2.60	1.29	2.88	1.37	−3.09**	−0.20	2.86	1.36	2.74	1.34	1.28	0.09
In this time when I have to stay at home, I spend many hours a day in the morning and / or afternoon playing video games or watching television	3.49	1.27	2.81	1.40	7.62**	0.50	3.20	1.34	2.97	1.41	2.44*	0.16
In this period in which I have to stay at home, I spend much more time than before on social media such as Instagram or Facebook	3.30	1.31	3.83	1.18	−2.74**	−0.43	3.71	1.26	3.62	1.30	0.99	0.07

*p < 0.05; **p < 0.01. M, mean; SD, standard deviation; g, Hedge’s g.

As for what the teenagers in the sample missed most in this time of restrictions, the majority of the participants stated that they especially missed being able to meet friends (55.8%) and relatives (62.5%) and staying out later in the evening due to the closure of premises such as restaurants, pubs and discos and the prohibition to go out except for reasons of absolute necessity (52%). To confirm this, a large majority of the sample said they found significant support from family (81.1%) and friends (65.3%) to face this time when they had to stay home.

### Psychological Experiences During Quarantine (Feeling, Mood, and Emotions)

The responses of the adolescents show heterogeneous psychological reactions to the experience of quarantine. To better investigate the specific emotion and feeling they perceived, the responses to some of the more relevant items were first examined. This analysis aimed to capture a snapshot of the emotional state of adolescents during the quarantine.

The majority of those interviewed stated that they stayed physically well (68.7%). Males felt better than females but the difference was small (males: *M* = 3.32, *SD* = 1.17; females: *M* = 2.97, *SD* = 1.16; *t* = 4.54, *p* < 0.001; *g* = 0.30). However, quarantine influenced their sense of security and self-confidence: 43.1% of the students reported feeling less secure than in the past. Females were less self-confident than males with a medium ES (females: *M* = 2.50, *SD* = 1.23; males: *M* = 3.18, *SD* = 1.14; *t* = 8.58, *p* < 0.001; *g* = 0.56) while there were no significant differences by age, the area in which the person lived, and other socio-demographic variables.

Concerning psychological status, about 40% of students reported feeling tenser and sadder (42.6%) and more irritable (49.6%) than usual, with increased ruminations (59.6%). A high percentage reported difficulty concentrating (55.9%) and sleeping (55.6%). However, only a small percentage of the students reported difficulties eating, such as forgetting to eat or skipping meals (13.7%), disturbances in heartbeat (18.7%), crying frequently (34.4%), or other symptoms that showed a clear condition of pathological stress. According to the t-test results, females and adolescents living in a red zone tended to have more significant difficulties in this regard, as shown in Table 5.

Interestingly, the responses of the students showed their great empathy and interest in socialization. A high percentage of respondents said they were not bored listening to others’ problems (46.7%) and reported being able to manifest their emotions (41.5%). These results agree with the findings discussed in the previous section, which showed that the things and situations students missed most were meeting friends, staying with relatives, and being out late in the evening.

Multiple regression analyses were performed to investigate the impact of sociodemographic and perceived health risk variables on the psychological outcomes (positive and negative feelings *z* scores). Sociodemographic variables, perceived health risk, and adherence to government restrictive measures were used as independent variables while positive and negative feelings *z* scores were the dependent variable. All regression assumptions were checked. There was linearity as assessed by partial regression plots and a plot of studentized residuals against the predicted values. There was independence of residuals, as assessed by a Durbin–Watson statistic of 1.9. There was homoscedasticity, as assessed by visual inspection of a plot of studentized residuals versus unstandardized predicted values. There was no evidence of multicollinearity, as assessed by tolerance values greater than 0.1. There were no studentized deleted residuals greater than ±3 standard deviations, no leverage values greater than 0.2, and values for Cook’s distance above 1. The assumption of normality was met, as assessed by a Q-Q Plot.

Regarding positive feelings, a significant regression equation was found (*F* = 6.995, *p* ≤ 0.001), with an *R square* of 0.11. More specifically, significant predictors of positive feelings were gender (*t* = −5.851, *p* < 0.001, *Std β* = −0.185), region (*t* = 2.326, *p* = 0.02, *Std β* = 0.074), confidence in the information received on COVID-19 (*t* = 2.631, *p* = 0.009, *Std β* = 0.084), perceived susceptibility (*t* = −2.386, *p* = 0.017, *Std β* = −0.089), ease in respecting government measures (*t* = 4.698, *p* < 0.001, *Std β* = 0.152), and belief that the government measures were justified (*t* = 2.480, *p* = 0.013, *Std β* = 0.082). According to these results, females reported less positive feelings than males on average as well as adolescents living in Northern Italy. Furthermore, higher confidence in the information received on COVID-19, higher perceived susceptibility, higher ease in respecting government measures and higher beliefs that these measures are justified were predictive of positive feelings. Table 6 shows the significant results of the regression analyses and the contribution of each predictor to the dependent variable.

**TABLE 6:** Multiple regression analyses of possible predictors for positive and negative psychological outcomes in the sample.

	*Std β*	*t*	*p*
***Negative Feelings F = 11.103; p ≤ 0.001; R square = 0.16***			
Gender	0.284	9.291	<0.001
Age	0.119	3.900	<0.001
Living in a “red zone”	0.090	2.905	0.004
Perceived seriousness	0.085	2.690	0.007
Fear of getting COVID-19	0.091	2.809	0.005
Compliance with Government measures	−0.103	−3.281	0.001
***Positive Feelings F = 6.995; p < 0.001; R square = 0.11***			
Gender	−0.185	−5.851	<0.001
Region	0.074	2.326	0.020
Confidence in information on COVID-19	0.084	2.631	0.009
Perceived susceptibility	−0.089	−2.386	0.017
Compliance with Government measures	0.152	4.698	<0.001
Beliefs that restrictions are right	0.082	2.480	0.013

Regarding negative feelings, the regression model was significant (*F* = 11.103, *p* < 0.001), with an *R square* of 0.16. More in detail, significant predictors of negative feelings were gender (*t* = 9.291, *p* < 0.001, *Std β* = 0.284), age (*t* = 3.900, *p* < 0.001, *Std β* = 0.119), living in a red zone (*t* = 2.905, *p* = 0.004, *Std β* = 0.090), perceived seriousness (*t* = 2.690, *p* = 0.007, *Std β* = 0.085), fear of getting COVID-19 (*t* = 2.809, *p* = 0.005, *Std β* = 0.091), and compliance with government measures (*t* = −3.281, *p* = 0.001, *Std β* = 0.152). According to these results, females and older adolescents reported more negative feelings than males and younger adolescents on average. Furthermore, living in a red zone, a higher perceived seriousness, a higher fear of getting COVID-19 and a lower compliance with government measures were predictive of negative feelings. Table 6 presents the significant results of the regression analyses and shows the contribution of each predictor to the dependent variable.

In summary, the model showed a moderate but significant impact of both the sociodemographic and the health risk perception variables related to COVID-19 experience on the perception of negative and positive feelings.

### Expectations for the Immediate Future

Participants’ expectations for the immediate future were also investigated through an open-ended question about how they imagined the upcoming summer holidays. Data were codified according to the modality described in the Procedure section. We first categorized the responses into 55 categories and then progressively reduced these categories. The adolescents were aware that the experience of quarantine would continue to produce effects during the summer period. In this regard, a significant percentage of the adolescents in the sample said that their summer would be “different” or would have “different limitations” (24.1%). Uncertainty and doubt were widespread feelings, as expressed by these answers: “My summer will be full of anguish, doubts, and perplexity, but my friends are enough for me to feel good”; “I am very worried because I cannot imagine how it will be but I look forward to it as much as every year.”

In addition, 26.3% of the sample thought that summer would be sad, boring, or horrible, as demonstrated by the following quotes: “If transportation does not reopen, my summer will be wasted, the collapse of different dreams and projects that have so far pushed me to go on and resist a very dense pool of mud in which I will struggle so much not to sink”; “Like a prison, locked between the walls and between the screams, as if it were winter or autumn, without being able to see the seawater, which is freezing or boiling.” In some cases, quarantine only highlighted pre-existing difficult situations, such as in this case: “It will be the usual summer in which it is amplified that I have no friends and that no one ever invites me to go out and I will spend the day in my pajamas eating food at will and watching Netflix and Sky until I vomit.”

However, 8.9% thought summer would still be interesting and fun: “Interesting, I have high school exams, a girl, and too many friends to share my life with. I’m curious to see how everything will evolve.”

In general, the teenagers in the sample looked to the future with the hope of overcoming the difficult period of the pandemic and resuming a normal life, even if different from the previous one: “I don’t care what my summer will be like, I just hope that we will be able to get out of this situation with a new unitary spirit, I hope people understand that we are one family in one house”; “Meeting again, with the necessary restrictions, my friends, my grandparents, will be difficult as if we had to learn to live in a different way from what we were used to until a couple of months ago. It will be exciting!”.

## Discussion

This study aimed to provide a general overview of the psychological experience of quarantine in a large sample of Italian adolescents. According to other recent studies conducted on this topic in the Italian population, the COVID-19 emergency was a very difficult experience from an emotional point of view, and several categories such as health professionals have undergone significant stress with a consequent negative impact on their psychological well-being (Ramaci et al., 2020). To the best of the knowledge, this is the first study investigating the experience of the COVID-19 pandemics in Italian adolescents and with such a large number of subjects.

First, the perceived seriousness and susceptibility to COVID-19 were evaluated, as well as the impact of sociodemographic variables on the perception of health risk. According to the study results, Italian adolescents had a low perception of risk of COVID-19. Perceived comparative susceptibility and perceived seriousness in Italian adolescents were also very low. These results show that young people think that COVID-19 is not a potentially severe disease for them. Indeed, there is some evidence that young people are less vulnerable to the effects of the new coronavirus SARS-CoV-2 (Kolifarhood et al., 2020), although the possibility of getting the disease depends on the diffusion within the population. They underestimate the probability of getting the disease and show a very high trust in their good health, neglecting that the probability of being infected, albeit slight, is similar to that of their peers and people of other age groups.

Interestingly, as hypothesized, teenagers residing in a red zone reported higher perceived seriousness and susceptibility than those who did not reside in these zones. Furthermore, females showed a higher perceived seriousness than males. In both cases, the medium effect size suggests a role of these variables in influencing health risk perception. Therefore, living in an area with more restrictions than in other areas of the country may have contributed significantly to increase the perception of risk about the disease. Also, this information seems consistent with several studies demonstrating that women tend to have a higher perception of risk than men, thus avoiding risky behaviors to a greater extent (Harris et al., 2006).

Despite underestimating their risk of infection, however, the Italian teenagers who participated in this study were aware of the restriction measures necessary to contain the spread of the virus and they agreed with the limitations imposed by the government. These responses show high awareness of the potential danger of COVID-19 and acquire more value when considering that young people were conscious that they were not at serious risk, but that the risk was high for society as a whole.

The study also wanted to investigate the emotional and psychological impact of the quarantine period on the youth population in Italy. As underlined in the literature on this topic, prolonged school closure and home confinement during an epidemic can have a detrimental effect on children’s and adolescents’ physical and psychological well-being (Brooks et al., 2020; Wang et al., 2020). According to the results, this study also shows that Italian adolescents suffered the psychological effects of this quarantine period. Indeed, they had more marked negative feelings. More specifically, females and adolescents residing in the red zones with more restrictions showed higher levels of negative feelings related to the quarantine, in accordance with the study’s hypotheses. In this regard, effect sizes approaching to medium indicate a possible role of these variables in determining negative feelings in the adolescents of the sample, even if these feelings may be likely influenced also by other variables not considered in the study. However, it is essential to emphasize that these feelings are subjective perceptions rather than a psychopathological state. Indeed, quarantine did not reduce the empathy and sociability of young people. In this regard, the adolescents who participated in this study reported that they engaged in school activities remotely and carried out the assigned homework. Furthermore, they continued to listen to the problems of others and to express their emotions. No significant differences related to the regions where the teenagers lived were found. This is very interesting data, leading to hypothesize that the negative feelings reported by the participants may be more related to the adolescent period than to the pandemic itself.

The results also showed a moderate but significant impact of both the sociodemographic and the health risk perception variables related to the COVID-19 experience in the perception of negative and positive feelings. More specifically, being male, living in a region with less virus spread, reporting low levels of perceived susceptibility and high compliance and agreement with government measures were all variables associated with the perception of positive feelings. On the contrary, being female and older, living in a red zone, reporting high levels of perceived seriousness and fear of getting COVID-19, and being less compliant with government measures were associated with more negative feelings.

As recently underlined by Wang et al. (2020), the adverse effects on psychological well-being are more significant when children and adolescents are confined to their homes without the possibility of carrying out activities outside and meeting peers. In confirmation of these considerations, this study showed that the majority of teenagers interviewed suffered in particular from not being able to meet friends and relatives, as well as from not being able to go out and stay out late in the evening.

The results also confirmed the literature data demonstrating that when children and teenagers do not go to school and stay home, they are physically less active, are exposed to much more screen time, and have irregular sleep patterns. Similarly, the teenagers in the sample had difficulty falling asleep and spent more time watching television, playing video games, or using social networks. However, a significant percentage stated that they dedicated at least an hour a day to playing a musical instrument, dancing, exercising, acting, or drawing. In light of these insights, it is important to promote healthy habits and lifestyles in adolescents to reduce psychosocial stress and improve the psychological and physical well-being of the young population.

Finally, the quarantine experience was also associated with a widespread sense of uncertainty about the near future in the adolescents interviewed in this study. Although some participants were convinced that the virus would disappear during the summer, allowing a return to normal life, most believed that their near future would be unpredictable or different due to the various health and social distancing rules that must be respected. In this regard, it is important to support the youth population in addressing the uncertainties related to the period following the quarantine to ensure better adherence to the limitations that will have to be faced to avoid a new outbreak of the epidemic.

This study has some important strengths. As already underlined, this is one of the first studies conducted in Italy about the perceived seriousness and susceptibility for COVID-19 as well as the effects of school closures and home confinement on the physical and psychological well-being of adolescents. Another strength is certainly the large sample size, with almost 1000 Italian teenagers interviewed from North, Central, and South Italy.

However, there are also several limitations. First, this is a cross-sectional study so an exact causal relationship between the variables could not be established. Secondly, an internet-based questionnaire with self-reported measures was used, so it was not possible to ascertain the accuracy of the answers to the questions and the possible influence of self-report bias on the results. Finally, not all Italian regions are represented in the sample; however, it is representative of the three main areas in which Italy is generally divided (North, Central, and South).

## Conclusion

This study has several social and psychological implications. In particular, the results underline that the COVID-19 emergency has undoubtedly had a significant impact on the lifestyle and psychological well-being of Italian adolescents. In light of these findings, the physical and mental impact of the COVID-19 epidemic on children and adolescents is a matter of fundamental importance both for governments and families and cannot be neglected, especially in this phase of a progressive resumption of ordinary life. Therefore, it is necessary to prepare adequate strategies to support the youth population in addressing the uncertainty associated with the pandemic and the quarantine period to reduce the psychological impact of school closures and home confinement as much as possible and guarantee adequate support to deal with the return to school.

## Data Availability Statement

The raw data supporting the conclusions of this article will be made available by the authors, without undue reservation.

## Ethics Statement

The studies involving human participants were reviewed and approved by Chair of School and Family Psychology, DISFOR, University of Catania. Written informed consent to participate in this study was provided by the participants, and where necessary, the participants’ legal guardian/next of kin.

## Author Contributions

EC designed the study, analyzed the data, and wrote the first draft. VLLR revised the manuscript. Both authors contributed to the article and approved the submitted version.

## Conflict of Interest

The authors declare that the research was conducted in the absence of any commercial or financial relationships that could be construed as a potential conflict of interest.
